# Anti-Müllerian Hormone Recruits BMPR-IA in Immature Granulosa Cells

**DOI:** 10.1371/journal.pone.0081551

**Published:** 2013-11-28

**Authors:** Lauriane Sèdes, Arnaud Leclerc, Hadia Moindjie, Richard L. Cate, Jean-Yves Picard, Nathalie di Clemente, Soazik P. Jamin

**Affiliations:** 1 Inserm U782, Clamart, France; 2 Université Paris-Sud, UMR- S0782, Clamart, France; Institut Jacques Monod, France

## Abstract

Anti-Müllerian hormone (AMH) is a member of the TGF-β superfamily secreted by the gonads of both sexes. This hormone is primarily known for its role in the regression of the Müllerian ducts in male fetuses. In females, AMH is expressed in granulosa cells of developing follicles. Like other members of the TGF-β superfamily, AMH transduces its signal through two transmembrane serine/threonine kinase receptors including a well characterized type II receptor, AMHR-II. The complete signalling pathway of AMH involving Smads proteins and the type I receptor is well known in the Müllerian duct and in Sertoli and Leydig cells but not in granulosa cells. In addition, few AMH target genes have been identified in these cells. Finally, while several co-receptors have been reported for members of the TGF-β superfamily, none have been described for AMH. Here, we have shown that none of the Bone Morphogenetic Proteins (BMPs) co-receptors, Repulsive guidance molecules (RGMs), were essential for AMH signalling. We also demonstrated that the main Smad proteins used by AMH in granulosa cells were Smad 1 and Smad 5. Like for the other AMH target cells, the most important type I receptor for AMH in these cells was BMPR-IA. Finally, we have identified a new AMH target gene, *Id3*, which could be involved in the effects of AMH on the differentiation of granulosa cells and its other target cells.

## Introduction

Anti-Müllerian hormone (AMH) also called Müllerian inhibiting substance (MIS) is a member of the TGF-β superfamily. AMH is well known for its role in Müllerian duct regression in male fetuses [1]. In female fetuses, the lack of AMH expression allows the development of the Müllerian ducts into oviduct, uterus, cervix and the upper part of the vagina. Postnatally, AMH is secreted by granulosa cells (GCs) of small growing follicles (preantral and small antral) [1]. In mice, AMH is a negative regulator of the primordial to primary follicle transition [2]. In addition it decreases FSH sensitivity of growing follicles [3]. In clinics, serum AMH is now widely used in oncology and gynecology. Indeed, it is a very useful diagnostic and prognostic tool, as an early indicator of relapse of ovarian GC tumors [4] and as a reliable marker of the ovarian follicular status. AMH assay is also an important tool to control ovarian hyperstimulation [5]. However, despite the increasing interest of ovarian AMH in clinics, little is known on its mechanism of action on GCs.

Members of the TGF-β family signal through a type II transmembrane serine/threonine kinase receptor which forms a complex with a type I serine/threonine kinase receptor [6]. The type II receptor phosphorylates serine and threonine residues of type I receptor. Once activated, the type I receptor phosphorylates the receptor-regulated Smads (R-Smad) which interact with a common partner Smad4. The Smad complex accumulates into the nucleus and regulates target gene expression [7]. TGF-β and activins activate TbetaRI and ActR-IB type I receptors and R-Smads 2 and 3, whereas Bone Morphogenetic Proteins (BMPs) mediate their effects through ActR-IA, BMPR-IA and BMPR-IB type I receptors and R-Smads 1, 5 or 8. This canonical signalling pathway is regulated at different levels, in particular by co-receptors which amplify or antagonize TGF-β family members action.

AMH has a single specific type II receptor (AMHRII, also known as MISRII) [8], [9]. Indeed, *Amh* and *Amhr2* inactivation causes the same phenotype in males [10], a complete retention of an ectopic female reproductive tract, indicating that the AMH type II receptor is the only type II receptor that transduces AMH signal [11]. Moreover, in human, mutations of *AMH* or *AMHRII* are involved in Müllerian duct derivative persistance [12]. Regarding the other components of AMH signalling pathway, it was first shown in different cell lines of gonadal origin that AMH phosphorylates R-Smad 1/5/8, meaning that it uses ActR-IA, BMPR-IA or BMPR-IB type I receptors to transduce its effects [13]. Then the disruption of these type I receptors and R-Smads in mice led to the conclusion that BMPR-IA is the primary type I receptor required for Müllerian duct regression but that ActR-IA is capable of transducing the AMH signal in the absence of BMPR-IA, and that R-Smad 1/5/8 function redundantly [14]–[16]. Similarly, in the immature Sertoli cell line SMAT-1, AMH mediates its signal through BMPR-IA and ActR-IA [17]. In contrast, the type I receptors and R-Smads involved in AMH effects on post-natal GCs remain unknown. In addition, to date, no co-receptor has been found for AMH. Because AMH share with BMPs its type I receptors and R-Smad proteins, it could have the same co-receptors, like the Repulsive Guidance Molecule (RGM) [18] which were first shown to induce axonal guidance during neurogenesis [19] and have three isoforms: RGMa, RGMb (Dragon) and RGMc (Hemojuvelin).

To date, only few AMH target genes have been identified. In males, AMH inhibits Sertoli and Leydig cells differentiation through repression of several steroidogenic proteins like P450scc (*Cyp11a1*), 3β-HSD (*Hsd3b1*) or P450C17 (*Cyp17a1*) [17], [20], [21]. Little is known on the genes involved in the inhibitory effect of AMH on GCs differentiation, preventing the study of AMH mechanism of action on these cells. Aromatase (*Cyp19a1*) and LH receptor (*Lhcgr*) are down-regulated by AMH in rat and porcine GCs [22]. In human GCs, FSHR (*Fshr*) is also repressed by AMH [23] which could explain the inhibitory role of this hormone on follicles sensitivity to FSH. Because BMPs share their signalling pathway with AMH and regulate GCs differentiation, BMPs target genes involved in this process could also be modulated by AMH. This could be the case for the family of Inhibitor of differentiation/Deoxyribonucleic Acid-Binding (*Id*) genes which are BMP2 and BMP4 target genes in mice [24]. These genes have been shown recently to be related to the status of GC differentiation [25]. They lack a domain required for DNA binding and act as negative antagonists of bHLH transcription factors and gene expression in mammals [26].

In this study, we used primary GCs isolated from immature mice to identify target genes and co-receptors potentially implicated in AMH effects on GCs differentiation and to define the involvement of the different type I receptors and Smads proteins in this process. We also took advantage on conditional mutant mice for different actors of AMH signalling pathway to confirm our data. Our studies reveal that *Id3* is a new AMH target gene in GCs. In addition, this work indicates that BMPR-IA and Smad 1/5 are the main components of AMH signalling pathway in GCs.

## Materials and Methods

### Ethics Statement

Housing and care, method of euthanasia and experimental protocols were conducted in accordance with the recommendations of the French Accreditation of Laboratory Animal Care and in compliance with the NIH Guide for Care and Use of Laboratory Animals. The animal facility is licensed by the French Ministry of Agriculture (agreement N°C92-023-01). All animal experiments were supervised by Dr. Soazik Jamin (agreement delivered by the French Ministry of Agriculture for animal experiment N°92–299). Animals were sacrificed with CO_2_. All efforts were made to minimize animal suffering.

### Reagents

Recombinant human AMH was produced from the culture medium of Chinese hamster ovary (CHO) cells stably transfected with a human AMH cDNA, purified and cleaved by plasmin (plasmin-cleaved AMH or PC-AMH) as previously described [27]. PC-AMH subsequently called AMH was used at a concentration of 8 or 40 nM. BMP2 was a kind gift of Prof. Walter Sebald (University of Würzburg, Germany) and was used at a concentration of 10 nM [28]. TGF-β was purchased from R&D Systems (Lille, France) and used at 1 nM. Smad1-Gal4, Smad5-Gal4, Smad8-Gal4 and Gal-Luc plasmids were kindly provided by Dr. Azeddine Atfi (Inserm UMR S938, Paris).

### Mice

Immature female C57BL/6JRj mice (3 weeks old) were purchased at Elevage Janvier (Le Genest-St-Isle, France).

*Amhr2*-*cre* [15], *Acvr1* ^+/−^ [29], *Bmpr1a* ^+/−^ [30], *Acvr1* ^fx/fx^ [31], *Bmpr1a* ^fx/fx^ [32] mice were maintained on a C57BL/6J; 129/SvEv mixed genetic background. *Amhr2*-*cre*; *Acvr1* ^+/−^ or *Amhr2*-*cre*; *Bmpr1a* ^+/−^ males were bred to *Acvr1* ^fx/fx^ or *Bmpr1a* ^fx/fx^ females to generate females that were conditionally null for *Acvr1* or *Bmpr1a* respectively (designated as *Acvr1* cKO and *Bmpr1a* cKO).

### Mouse genotyping

Genomic DNA was extracted from tail biopsies using a NucleoSpin Tissue kit (Macherey-Nagel, Hoerdt, France) according to the manufacturer's instructions. The primers used to detect *Acvr1* and *Bmpr1a* alleles are listed Table S1. The amplification conditions were 95°C for 5 min, followed by 94°C for 45 s, 58°C for 45 s, and 72°C for 45 s (35 cycles), with a final extension at 72°C for 10 min. The amplified PCR fragments were analysed on 2% agarose gels.

### Primary cultures of mouse granulosa cells

The preparation of mouse GCs primary cultures was adapted from a rat primary GC culture protocol [33]. Immature ovaries from 3 weeks old mice were collected in RPMI medium (Invitrogen). Then, they were exposed to 6.8 mM EGTA, 0.2% BSA in Medium 199 (Invitrogen) for 15 min at 37°C. After a 5 min centrifugation at 500 rpm, ovaries were placed in a hypertonic solution (0.5 M sucrose, 1.8 mM EGTA, 0.2% BSA) in Medium 199 for 5 min at 37°C. Three volumes of Medium 199 were added to stop the reaction. After a 5 min centrifugation at 500 rpm, ovaries were placed in DMEM/F-12 medium with 1% Fetal Bovine Serum (FBS, Life technologies) and GCs were dissociated with a blunt spatula. Cells were pelleted by a 10 min centrifugation at 1300 rpm. Supernatant was discarded and cells were resuspended in DMEM/F-12 without phenol red, 10% FBS. The collected cells were counted in the presence of Trypan blue (0.07%) and seeded in DMEM/F-12 without phenol red, 10% FBS and 1% penicillin/streptomycin (Eurobio, Courtaboeuf, France) at 37°C in 5% CO2. 36 h after plating, GCs were exposed to AMH, BMP2 or TGF-β in DMEM/F-12 without phenol red, 1% FBS and 1% penicillin/streptomycin (Eurobio) for 24 h for RNA analyses. For protein analyses, GCs were starved in serum-free medium for 1 h prior to a 3 h AMH, BMP2 or TGF-β exposure.

### β-galactosidase and pmaxGFP transfection

Primary GCs seeded in 6-well plates (5×10^5^ cells/well) were transfected using Fugene6 reagent (Roche Diagnostics, Meylan, France) with 1 µg β-galactosidase reporter gene or pmaxGFP vector when cells were 80% confluent. After 24 h, β-galactosidase activity was detected with X-gal staining and GFP signal was directly visualized.

### Immunofluorescence

Cells were seeded in 4-well Lab-Tek (1×10^5^ cells/well) (Dutscher, Brumath, France) and incubated at 37°C in 5% CO2. After a quick wash in 1X phosphate-buffered saline (PBS), the cells were fixed in 4% paraformaldehyde (PFA) for 15 min. After a permeabilization step in tris-buffered saline (TBS)/0.2% Triton, cells were incubated in blocking buffer (PBS 1X, 10% BSA, 0.3% Triton) for 1 h at room temperature. Then cells were incubated with the anti-AMH (1∶500, Santa Cruz, Heidelberg, Germany) primary antibody in dilution buffer (PBS 1X, 1% BSA, 0.3% Triton) overnight at 4°C. After one wash in TBS/Triton, cells were incubated with the secondary antibody (Alexa Fluor-488 conjugated rabbit antibody) 1 h at room temperature in the dark. Cells were washed in high salt PBS and slides were covered with mounting medium containing DAPI (Vector Laboratories, Abcys, Les Ulis, France).

### Immunohistochemistry

Ovaries from 3 weeks old C57BL/6J females were collected and fixed in 4% PFA for 4 h at 4°C. Samples were then washed with PBS (pH 7.4), dehydrated in graded ethanol, embedded in paraffin and cut into 5 µm thick sections. For antigen retrieval, samples were boiled in citrate buffer 10 mM pH 6.0 at 80°C for 45 min. Thereafter, slides were washed with PBS and blocked with PBS-BSA 10% for 1 h before an overnight incubation with a primary anti-RGMb antibody (1∶100 in DAKO buffer, Santa Cruz). Specimens were washed with PBS and incubated with a secondary biotinylated antibody (anti-rabbit 1∶500) 1 h at room temperature. After washing, slides were incubated 1 h in ABC reagent (ABC kit, Vectastain, Abcys) and stained with DAB reagent during 5 min.

### siRNA gene knockdown

Small-interfering RNAs (siRNAs) for *Acvr1* (59932), *Bmpr1a* (160872), *Bmpr1b* (60258), and a negative control siRNA #1 were obtained from Ambion (Life Technologies) and siRNA *Rgmb* (J05553408) was obtained from Dharmacon (Fisher Scientific, Illkirch, France). siRNAs were used at a final concentration of 100 nM. Primary GCs seeded in 6-well plates (5×10^5^ cells/well) were transfected using Oligofectamine reagent (Life Technologies) with 100 nM siRNA when cells were 50% to 80% confluent. The medium was removed after 3 h and replaced by DMEM/F-12 1% FBS. The cells were then cultured 3 h or 24 h in the presence of 8 nM AMH.

siRNA GAPDH-cy3 (4390849, Life Technologies) used to show transfection efficiency was transfected as described above and the fluorescence was analysed by flow cytometry (FACS Calibur, Becton Dickinson).

### Luciferase Assay

Primary GCs seeded in 6-well plates (5×10^5^ cells/well) were co-transfected with a luciferase reporter (UAS-luc, 300 ng), expression construct (Smad-Gal4, 200 ng) [34] and pRLTK (1 ng, Promega) as a control for transfection efficiency. Transfection was performed using FugeneHD according to the manufacturer's instructions (Roche). Cells were subsequently treated 24 h with AMH (8 nM), washed with PBS and lysed in passive lysis buffer (Promega). All lysates were analysed for Firefly and Renilla luciferase activity according to the manufacturer (Dual Luciferase kit, Promega). Results were expressed as a percentage of stimulation of Firefly luciferase activity (after normalization to Renilla luciferase activity) in the presence of AMH compared to cells cultured in control medium.

### RNA isolation and Reverse Transcription

Total RNAs were isolated using the RNeasy Minikit (QIAGEN) according to the manufacturer's instructions. Reverse transcription was performed in a total of 20 µl with the Omniscript Reverse Transcription Kit for RT-PCR (QIAGEN) using 500 ng or 1 µg total RNA, Omniscript reverse transcriptase, oligo-dT primers (1 µM) and random hexamers (10 µM) as recommended by the manufacturer. The samples were incubated 1 h at 37°C.

### PCR amplification

cDNA from primary GCs or total ovary were used to amplify *Inha*, *Lhcgr*, and *Fshr* by PCR. The primer pairs used are shown Table S2. PCR was performed using PCR mix (Qiagen). The amplification conditions were 95°C for 5 min, followed by 35 cycles of 94°C for 45 s, 58°C for 45 s, and 72°C for 45 s, with a final extension at 72°C for 10 min. The amplified PCR fragments were analysed on 2% agarose gels.

### Quantitative real-time PCR

Quantification of the content of *Amhr2*, *Acvr1*, *Bmpr1a*, *Bmpr1b, Cyp11a1, Fshr, Id3, Inha, Rgma*, *Rgmb*, *Rgmc, Smad1, Smad4, Smad5, Smad8*, *Star* and *Hprt* mRNA was performed by real-time PCR using the TaqMan PCR method. The primers and the UPL probes (Roche Diagnostics, Mannheim, Germany) used to amplify these genes are indicated Table S2. Real-time PCR was performed with one fifth dilution of the cDNAs using the Lightcycler 480 Probes Master kit (Roche Diagnostics, Mannheim, Germany). Primers were used at a concentration of 5 µM and probes at 1 µM. The PCR protocol used an initial denaturating step at 95°C for 10 min followed by 45 cycles of 95°C for 10 s, 58°C for 30 s, 72°C for 1 s. To generate standard curves, different concentrations of the purified and quantified PCR products were amplified. Relative gene expression was normalized to an endogenous control gene (*Hprt*).

### Western blot

Mouse primary GCs were seeded into 6 wells plates at a density of 5×10^5^ cells/well in 2ml of culture medium. Cells were then harvested, and lysed in 50 mM Tris HCL (pH 7.4)/150 mM NaCl, 1% protease inhibiting cocktail and 1% Triton. Insoluble material was removed by centrifugation at 12,000×g, for 5 min at 4°C. The supernatants were recovered, and protein concentrations were measured using the BCA protein assay kit (Pierce). Equivalent amounts of protein lysates (8 to 20 µg) were subjected to 4–20% SDS-PAGE (Biorad) and electrophoretically transferred onto nitrocellulose membranes. After the blocking of non-specific binding sites for 1 h in Tris-buffered saline (25 mM Tris and 150 mM NaCl, pH 7.6) containing 5% non-fat milk and 0.1% Tween 20, the membranes were exposed to the primary antibodies (anti-phospho-Smad 1/5/8 and anti-phospho-Smad2/3 (Cell Signalling Technology) both at 1∶500) overnight at 4°C. Reactive proteins were detected with horseradish peroxidase-conjugated secondary antibodies (1∶5000) for 1 h at room temperature and developed with West Pico Western blotting detection reagents (Pierce). The membranes were stripped with a stripping buffer (Pierce), then reprobed with a mouse monoclonal antibody to β-Actin (Sigma Aldrich) or mouse monoclonal anti-α-Tubulin (Sigma Aldrich). Western blots were quantified using ImageJ software.

### Statistical analysis

All experimental data are presented as means ± SEM. Data were analyzed using *t*-test or one-way ANOVA followed by Tukey test for all-pair comparisons to compare respectively two or several means. A difference was considered statistically significant when the p-value was <0.05. * *p*<0.05, ** *p*<0.01, *** *p*<0.001. All calculations were made using GraphPad Prism 5.1 (GraphPad Software, La Jolla, CA).

## Results

### Primary cultures of immature mouse granulosa cells

**Figure 1:**
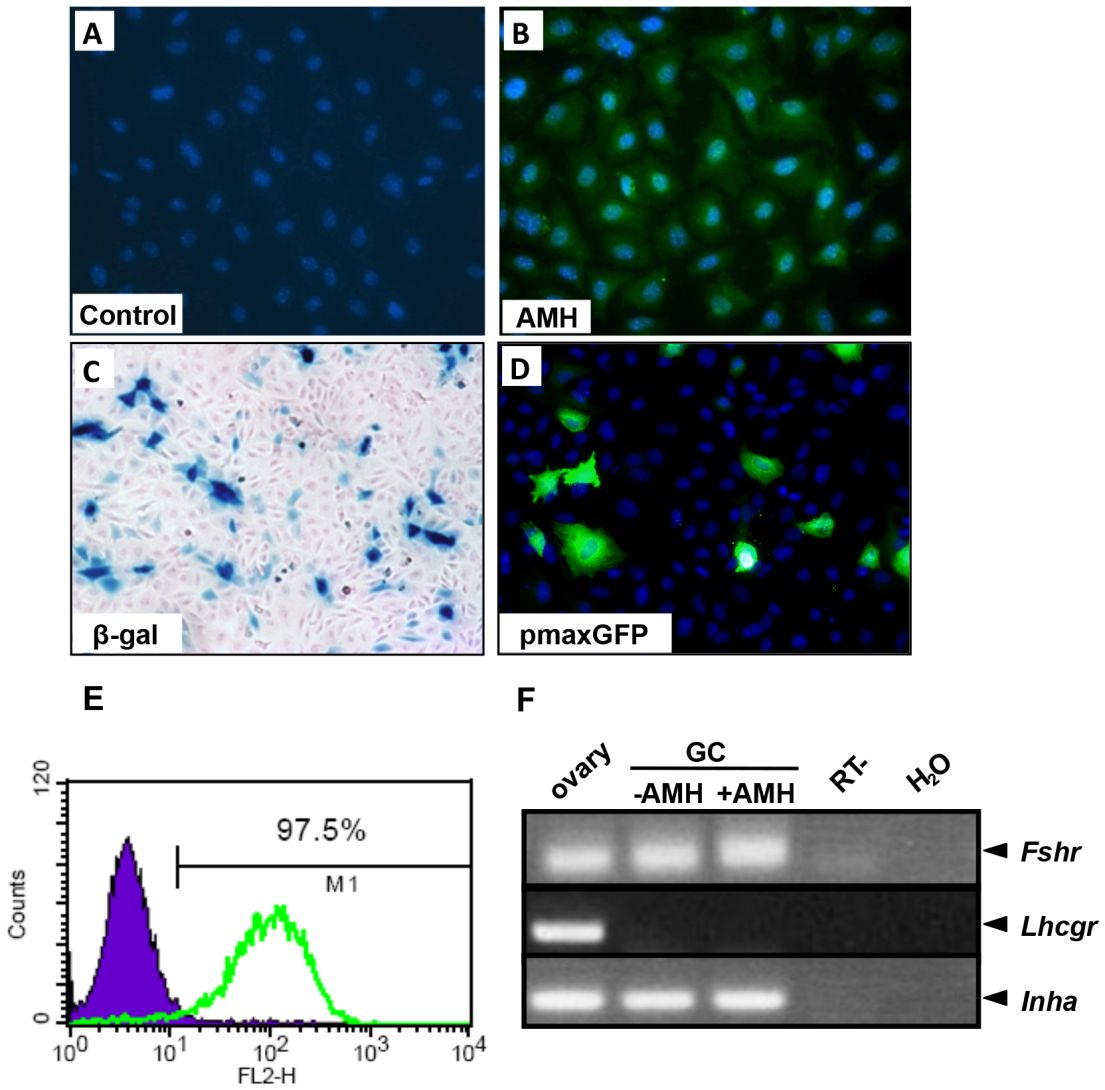
Characterization of granulosa cells in primary culture. Granulosa cells (GCs) were collected from 3 weeks old C57BL/6 mice ovaries and seeded at a density 1×10^5^ cells/well. 24 h later, GCs were incubated without primary antibodies (IgG) for the control condition (A) or with an anti-AMH antibody (B). The secondary antibody was coupled to FITC and DAPI was used to visualize the nucleus. AMH expression was detected in the cytoplasm as expected. To asssay the transfection efficiency, primary GCs were transfected either with a β-galactosidase vector (C, 1 µg), pMax-GFP vector (D, 1 µg) or GAPDH-cy3 siRNA (E). After 24 h, GC were fixed, stained with X-Gal and counterstained with nuclear fast red (C) or fluorescence was visualized (D). Alternatively, siRNA transfection efficiency was assayed with a GAPDH-cy3 siRNA on isolated GCs using flow cytometry analysis (E). (F), Markers of immature GCs were analysed by RT-PCR to confirm their status.

To identify the different components of the AMH signalling pathway in GCs, we first isolated immature GCs from 3 weeks old mice [33] and we characterized them for different criteria. Immature ovaries are mainly composed of small growing follicles whose GCs express AMH [35]. Therefore, we used an AMH antibody coupled to an Alexa fluor-488 secondary antibody to evaluate the purity of the primary GCs by immunofluorescence (Fig. 1A and 1B). About 90% of the cells expressed AMH which indicated an efficient enrichment of GCs (Fig. 1A and 1B). Since we had to transfect plasmid DNA in GCs, we then assayed transfection efficiency in these cells using a β-galactosidase reporter gene (Fig. 1C) or pMax-GFP vector (Fig. 1D). After X-Gal staining, we found that about 20% of the cells stained positively for β-galactosidase activity, indicating that they had been properly transfected (Fig. 1C). The transfection capacity of the primary GC culture was confirmed with pMax-GFP transfection by visualizing GFP-expressing cells (Fig. 1D). We also checked siRNA transfection efficiency. GCs were transfected with a fluorescently-labelled control siRNA (GAPDH-cy3 siRNA). The next day, the cells were trypsinized and the cell suspension was analysed by flow cytometry. We found that 97.5% of the GCs were transfected with the fluorescent siRNA, demonstrating a high siRNA transfection efficiency (Fig. 1E). We then checked for the presence of GCs markers (Fig. 1F). As in the total ovary, GCs expressed FSH receptor (*Fshr*) and α inhibin (*Inha*) (Fig. 1F). On the other hand, LH receptor (*Lhcgr*), a theca cell marker at this stage, was detected in the 3 weeks old ovary, as expected, but absent in the GCs. These results confirmed that the culture was at least 90% pure with little or no theca cell contamination.

### Granulosa cells can respond to plasmin cleaved AMH

**Figure 2:**
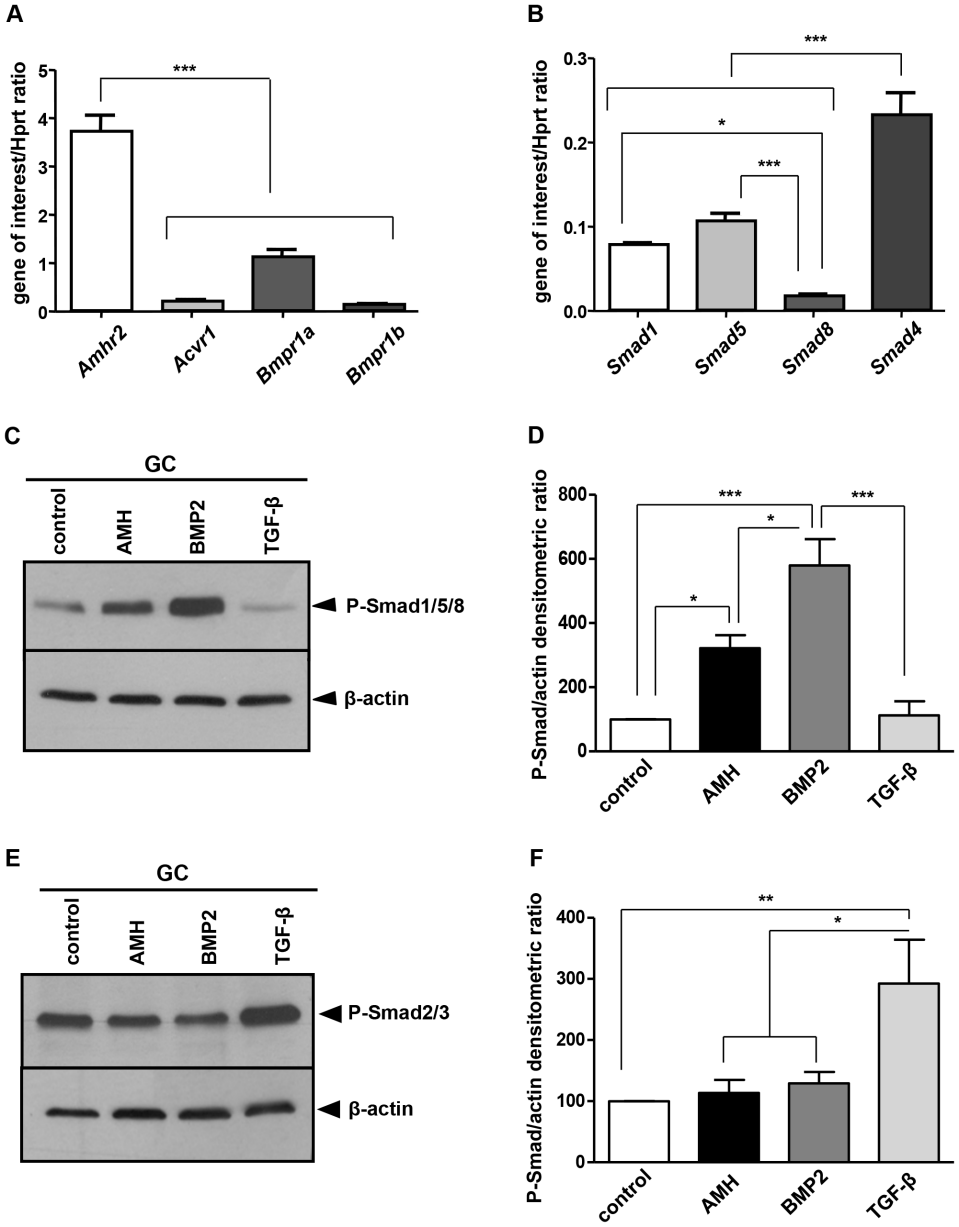
AMH activates Smad1 pathway in granulosa cells. GCs were isolated from 3 weeks old mice ovaries and seeded at 5×10^5^ cells/well in 6 wells plates. RNA was extracted to test AMH signalling pathway actors expression. The main known actors were analysed by real time PCR, including AMH type II and type I receptors (A, n = 6) and Smads proteins (B, n = 6). Data were analyzed using one-way ANOVA followed by Tukey test for all-pair comparisons. * *p*<0.05, ** *p*<0.01, *** *p*<0.001. GCs were exposed or not to 8 nM AMH, 10 nM BMP2 and 1 nM TGF-β. Proteins were extracted and analysed by Western blot using a phospho-Smad1/5/8 antibody (C, n = 4) or a phospho-Smad2/3 antibody (E, n = 4). Western blots were quantified and normalized to actin levels (D, F, n = 4). AMH could only activate the Smad1/5/8 pathway in GCs (C, D) but not the Smad2/3 one's (E, F). As controls, BMP2 only phosphorylated the Smad1/5/8 proteins (C, D) while TGF-β activated exclusively the Smad2/3 pathway (E, F). Data were analyzed using one-way ANOVA followed by Tukey test for all-pair comparisons. * *p*<0.05, ** *p*<0.01, *** *p*<0.001

We next studied the expression of the different components of AMH signalling pathway in GCs. Using real time PCR, we observed that all of the serine/threonine kinase receptors candidate genes (*Acvr1*, *Bmpr1a*, *Bmpr1b* and *Amhr2*) (Fig. 2A) and the three candidate *Smads* (Fig. 2B) were expressed in primary GCs. The results showed that *Amhr2* was expressed at higher levels (between 3.5 and 37 times) than the type I receptors (Fig. 2A), *Bmpr1a* being the most represented (Fig. 2A). Among Smads, *Smad4* was the most expressed, the levels of *Smad1* and *Smad5* were similar and *Smad8* protein was poorly represented (Fig. 2B). We then tested if primary GCs were responsive to AMH (plasmin-cleaved AMH). We analysed the response of GCs after a 3 h exposure to AMH by Western blot using a phospho-Smad 1/5/8 antibody (Fig. 2C). Both AMH and BMP2, which is the primary inducer, could increase phospho-Smad 1/5/8 levels (Fig. 2C and 2D) while TGF-β had no effect on this pathway. We then checked if AMH could also activate the alternative Smad2/3 pathway. TGF-β which is a primary inducer of the Smad 2/3 pathway was used as a positive control in parallel with AMH (Fig. 2E). As expected, in the presence of TGF-β, phospho-Smad 2/3 levels were increased in primary GCs (Fig. 2E and 2F). However, neither AMH nor BMP2 were able to activate the Smad2/3 pathway (Fig. 2E and 2F). Thus, the primary GCs displayed all the characteristics necessary for our study. They expressed all of the factors of the AMH signalling pathway, they were properly transfected and they were sensitive to AMH. These primary GCs were then used for the rest of the studies described in this paper.

### AMH target genes in granulosa cells

**Figure 3:**
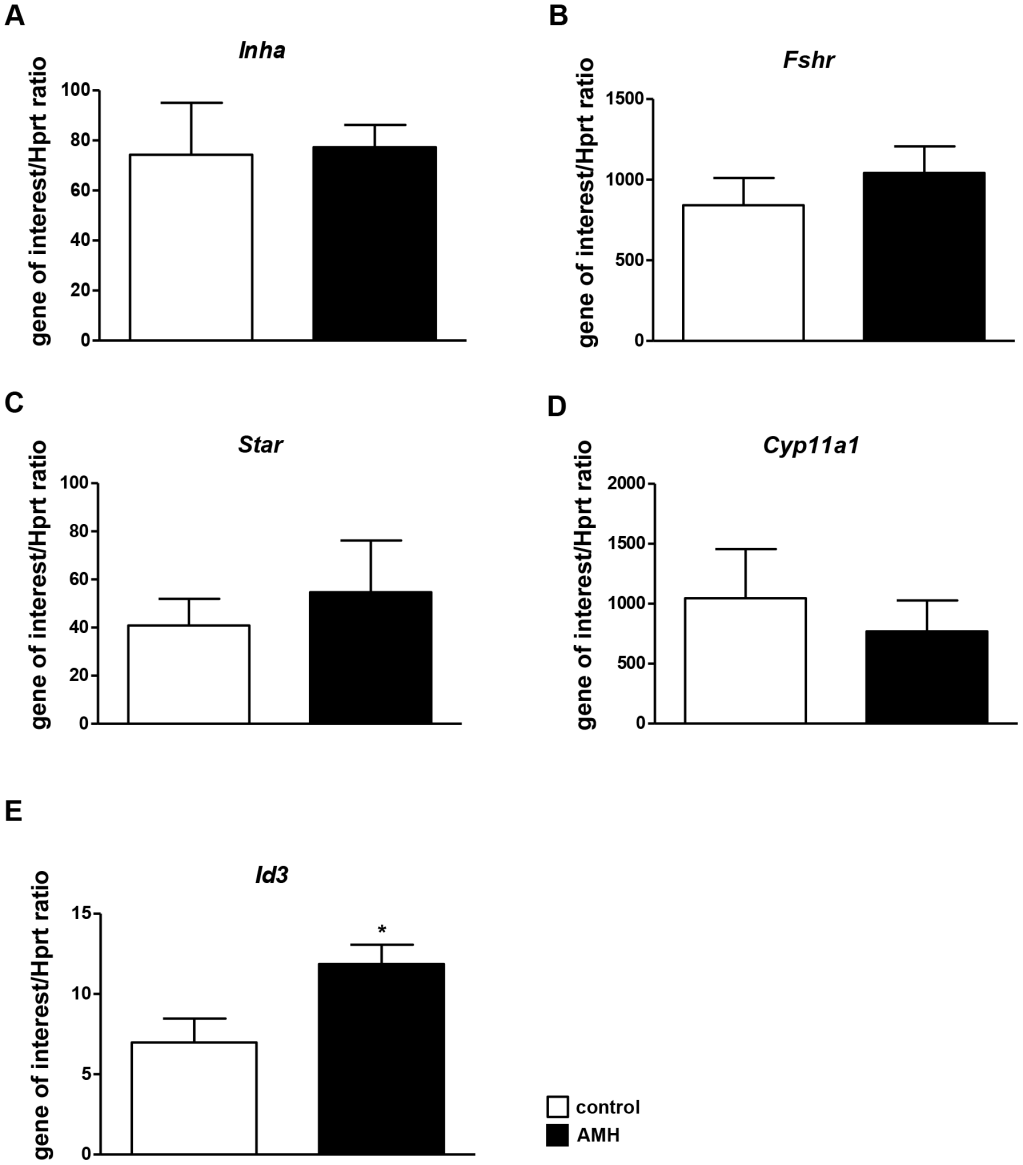
AMH target genes in granulosa cells. After collecting and seeding, GCs were exposed (▪) or not (□) to 8 nM AMH (A–E) for 24 h. The effect of AMH stimulation on different potential target genes was examined by real time PCR (A–D, n = 4; E, n = 5). Hprt expression was used to normalize the results. Data were analyzed using paired *t*-test. * *p*<0.05. *Id3* expression is significantly increased after AMH exposure.

To monitor AMH signalling, we then screened for downstream target genes. GCs were treated with AMH (8 nM) during 24 h, and the expression of candidate genes was analysed by real time PCR. We first tested AMH target genes previously identified in the ovary. Aromatase (*Cyp19a1*) and LH receptor (*Lhcgr*) were expressed at very low levels and we were not able to detect any variation of their expression after AMH exposure (data not shown). We then studied α inhibin (*Inha*) (Fig. 3A) and FSH receptor (*Fshr*) (Fig. 3B) whose expression was not modulated by AMH in GCs. Because AMH regulates genes encoding steroidogenic enzymes in Sertoli and Leydig cells, we tested whether their expression was also affected by AMH in GCs. 3 β-HSD (*Hsd3b1*) expression was too low to detect any variation (data not shown). *Star* (Fig. 3C) and P450scc (*Cyp11a1*) (Fig. 3D) were not regulated by AMH. Finally, we tested the effect of AMH on *Id* genes expression which are known to be up regulated by BMPs. *Id1, 2* and *3* were up-regulated by BMP2 in GCs but not by TGF-β (data not shown). Similarly to BMP2, AMH increased all *Id* genes expression but only *Id3* gene was significantly up-regulated (Fig. 3E). After 24 h in the presence of AMH, *Id3* expression in GCs is increased by 50%. Therefore, *Id3* gene was used in this study to assay siRNA knockdown experiments.

### Involvement of the different serine/threonine kinase receptors in AMH signalling

**Figure 4:**
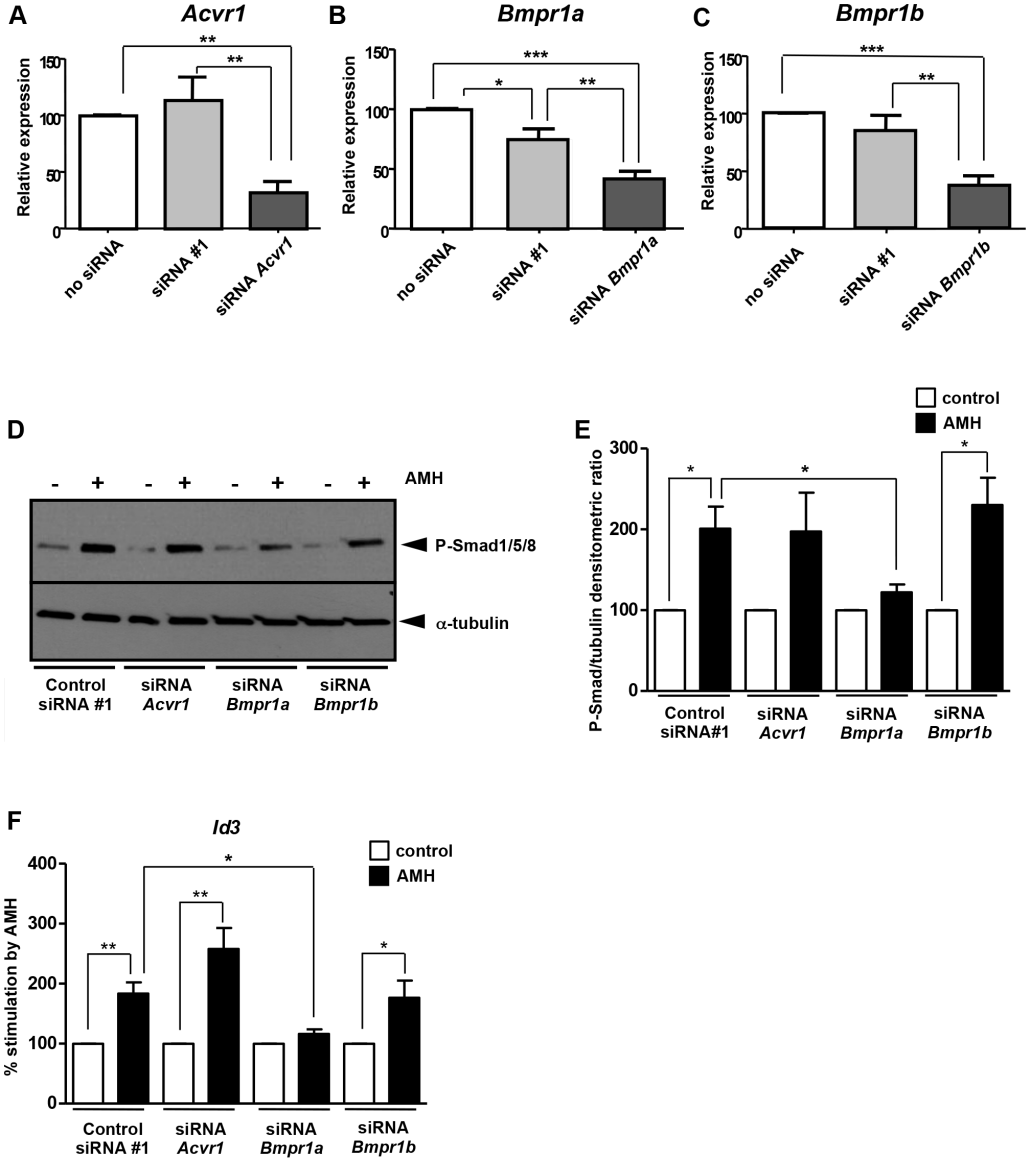
Involvement of serine/threonine kinase type I receptors. siRNA transfection for each type I receptor gene was performed when cells were 50% to 80% confluent. 24 h later GCs were exposed (▪) or not (□) to 8 nM AMH during another 24 h. The effect of siRNA on target gene expression was determined by real time PCR (A–C, n = 4). Data were analyzed using ANOVA followed by Tukey test for all-pair comparisons. The effect of *Acvr1*, *Bmpr1a* and *Bmpr1b* knockdown on AMH sensitivity was analysed by Western blot using a phospho-Smad1/5/8 antibody (D, n = 4) and was quantified and normalized (E). The effect of *Acvr1*, *Bmpr1a* and *Bmpr1b* knockdown on *Id3* expression was analyzed by real-time PCR (F, n = 3). Data were analyzed using paired *t*-test. * *p*<0.05, ** *p*<0.01, *** *p*<0.001. Only GCs transfected with siRNA against *Bmpr1a* present a significant decrease of AMH response.

To determine which type I serine/threonine kinase receptor(s) was important for AMH signalling pathway, we used siRNA for gene knockdown (Fig. 4). Primary GCs were isolated from immature ovaries and transfected when 50 to 80% confluence was reached with different siRNAs against *Acvr1*, *Bmpr1a* and *Bmpr1b*. After another 24 h of culture, the cells were exposed to AMH and total RNA were extracted 24 h later. We first checked the expression of each serine/threonine kinase type I receptor gene after siRNA transfection using real time PCR (Fig. 4A–C). For each type I receptor gene, we tested three different siRNAs (data not shown) and selected the most efficient one for the rest of the study. siRNA transfection led to a 80% decrease of *Acvr1* mRNA levels (Fig. 4A), a 70% decrease for *Bmpr1a* (Fig. 4B) and a 70% decrease for *Bmpr1b* (Fig. 4C). Since the down regulation was significant for each of the genes, we then analysed the responsiveness of these knocked-down GCs to AMH by Western blot with a phospho-Smad1/5/8 antibody (Fig. 4D). In parallel, the GCs were transfected with a negative control siRNA which does not interfere with any of the targeted RNAs. As expected, the negative control siRNA had no effect since the transfected cells could respond to AMH through the activation of the Smad1 pathway (Fig. 4D and 4E). Similarly, the *Acvr1* and *Bmpr1b* knockdown cells were sensitive to AMH. However, the effect was not significant for *Acvr1* because of the large standard deviation. On the other hand, in presence of siRNA against *Bmpr1a*, the effect of AMH on phospho-Smad1/5/8 levels was reduced significantly compared to GC transfected with the control siRNA. This result indicates that BMPR-IA is important for AMH signalling in GCs (Fig. 4D and 4E). We then analysed the effect of AMH on *Id3* expression in GCs transfected with the different siRNAs (Fig. 4F). *Id3* expression was up-regulated by 83% after AMH exposure in GCs transfected with control siRNA. *Id3* was also up-regulated by 158% in GCs transfected with siRNA against *Acvr1* and by 76% in GCs transfected with *Bmpr1b* siRNA. In contrast, in GCs transfected with siRNA against *Bmpr1a*, AMH was unable to up-regulate the expression of *Id3*. siRNA technology allowed us to show that BMPR-IA is important to transduce AMH signal in GCs (Fig. 4D, 4E and 4F).

### Granulosa cells from *Bmpr1a* cKO mice no longer transduce AMH signalling

**Figure 5:**
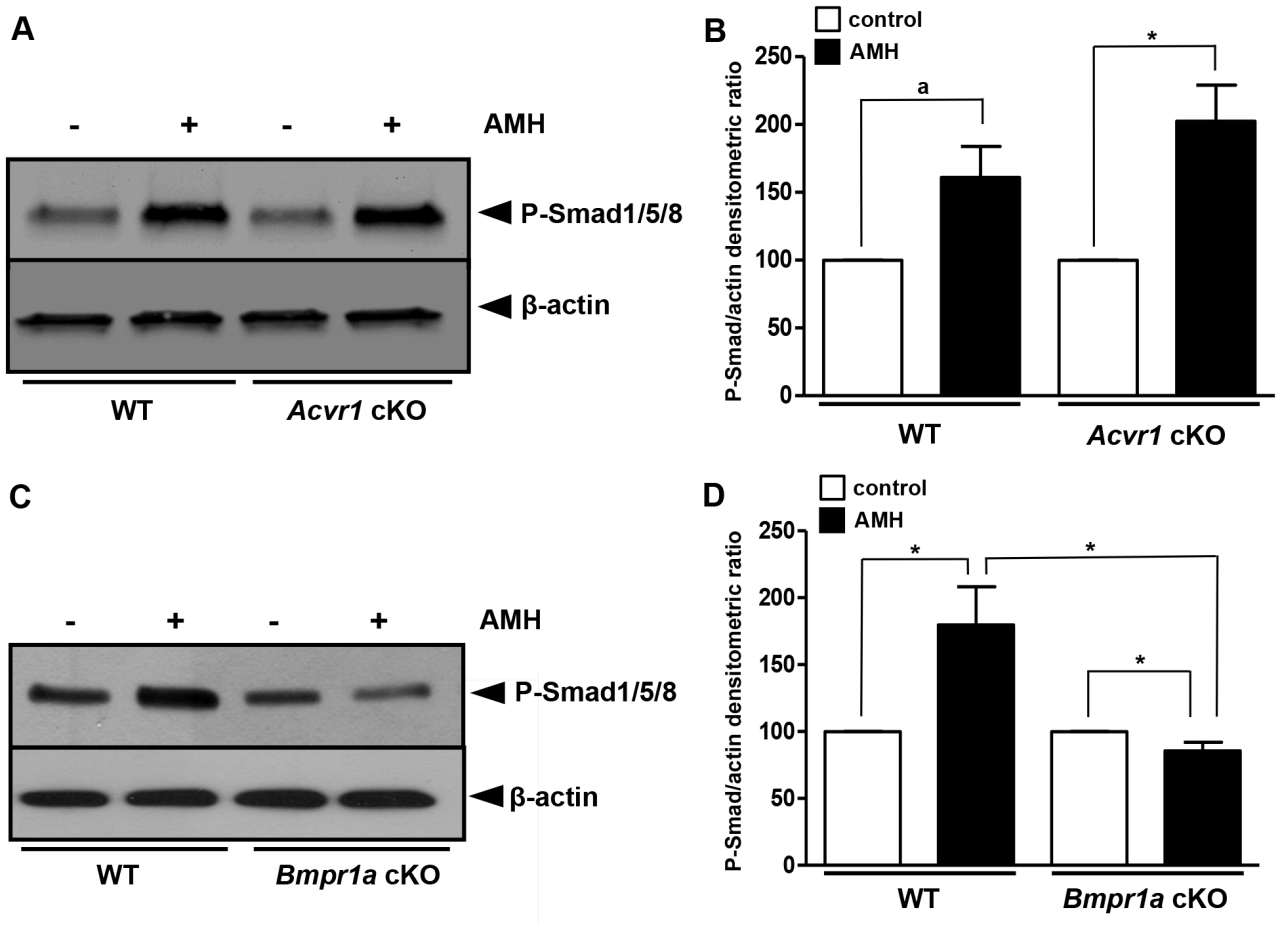
Granulosa cells from *Bmpr1a* cKO mice do not respond to AMH. *Amhr2-Cre*; *Acvr1* ^+/−^ or *Amhr2-Cre*; *Bmpr1a* ^+/−^ males were bred to *Acvr1* ^fx/fx^ or *Bmpr1a* ^fx/fx^ females to generate females that were conditionally null for *Acvr1* (A, n = 3) or *Bmpr1a* (B, n = 7) in GCs. GCs were exposed (▪) or not (□) to 8 nM AMH. The AMH response was tested in GCs from these cKO mice by Western blot using a phospho-Smad1/5/8 antibody (A,C). Western blots were quantified and normalized to actin levels (B n = 3, D n = 7). AMH induced the phosphorylation of Smad1/5/8 in GCs from *Acvr1* cKO (A, B) mice but not in those from *Bmpr1a* cKO mice (C, D). Data were analyzed using paired *t*-test. * *p*<0.05, a: *p* = 0.058. Only *Bmpr1a* conditional mutant GCs present a significant decrease of AMH response.

To confirm the siRNA results, we generated *Acvr1* and *Bmpr1a* conditional knockout (cKO) mice, using *Amhr2*-*cre* line to delete these genes in GCs. *Amhr2* ^+/*cre*^; *Acvr1* ^+/−^ or *Amhr2* ^+/*cre*^; *Bmpr1a* ^+/−^ males were bred to *Acvr1* ^fx/fx^ or *Bmpr1a* ^fx/fx^ females to generate females that were conditionally null either for *Acvr1* or *Bmpr1a* in GCs. *Amhr2* ^+/cre^; *Acvr1* ^fx/−^ and *Amhr2* ^+/cre^; *Bmpr1a* ^fx/−^ are designated *Acvr1* cKO and *Bmpr1a* cKO, respectively. GCs were isolated from these cKO mice ovaries and we tested the response of these cells to AMH by Western blot using a phospho-Smad 1/5/8 antibody. GCs from *Acvr1* cKO mice were as sensitive to AMH as GCs from control mice (Fig. 5A and 5B). In contrast, GCs from *Bmpr1a* cKO mice had lost their capacity to respond to AMH (Fig. 5C and 5D). The effect of AMH on phospho-Smad1/5/8 level was reduced significantly in GCs from *Bmpr1a* cKO compared to WT GCs (Fig. 5D). These results were consistent with the siRNA experiments and indicated that BMPR-IA was essential for AMH signalling in GCs.

### RGMb is not required for AMH signalling in granulosa cells

**Figure 6:**
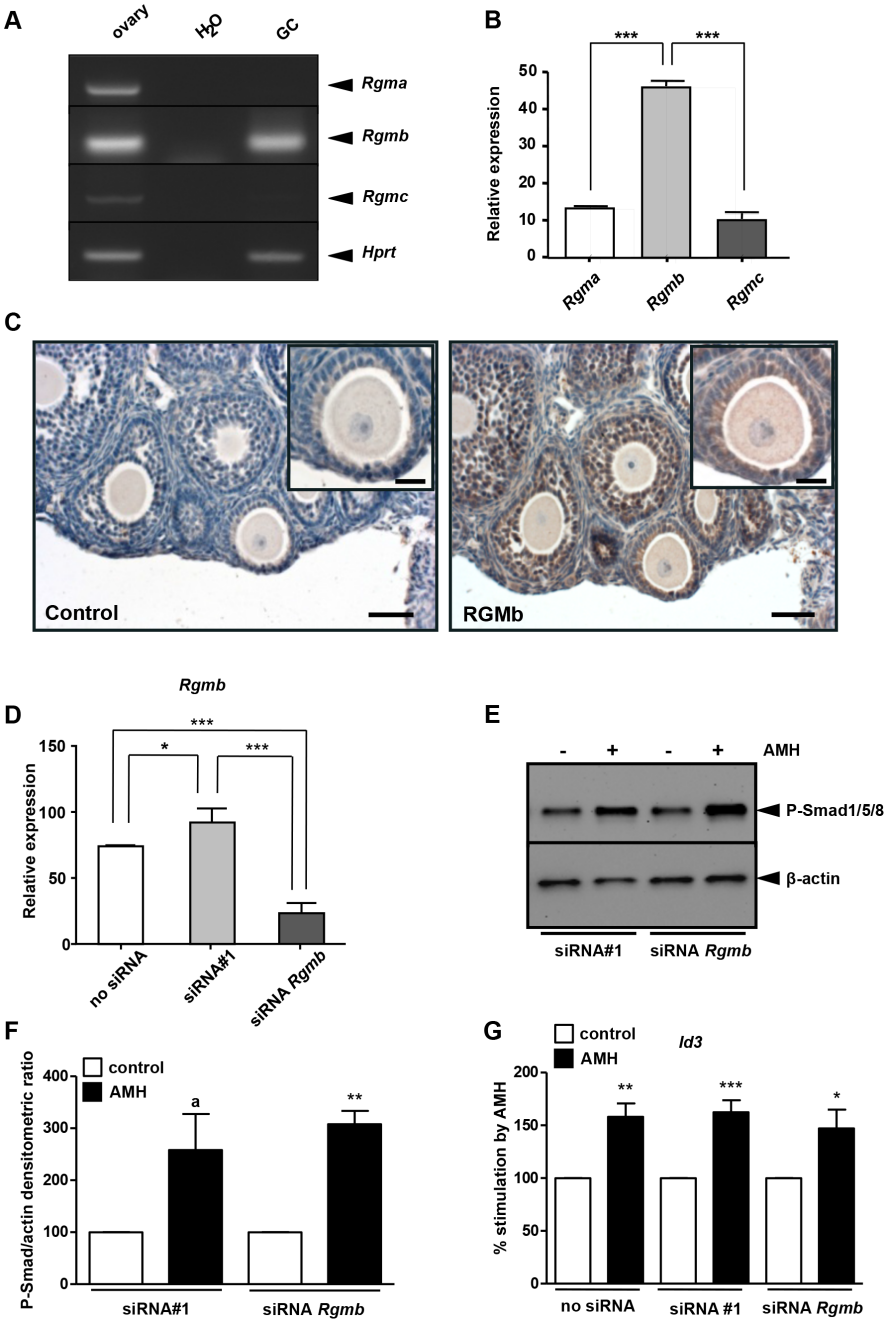
RGMb is not essential for AMH signalling in granulosa cells. *Rgma*, *b* and *c* expression was analysed by RT-PCR (A) or real time PCR (B, n = 6). (A) RT-PCR showed that all three *Rgm* were expressed in the mouse immature ovary (left lane) while *Rgmb* seemed predominantly expressed in GCs (right lane) (A). Real-time PCR confirmed that *Rgmb* was more expressed than *Rgma* and *Rgmc* in GCs (B). Data were analyzed using one-way ANOVA followed by Tukey test for all-pair comparisons. Mouse immature ovary was subjected to immunohistochemistry using an RGMb antibody (C). The left panel was the control without the primary antibody. The right panel shows that RGMb is expressed in the cytoplasm of granulosa cells (scale bar = 50 µm; insert, scale bar = 20 µm). siRNA transfection targeting *Rgmb* was performed when cells were 50% to 80% confluent (D–G). GCs were exposed (▪) or not (□) to 8 nM AMH. Real-time PCR was used to quantify the decrease in *Rgmb* expression. *Rgmb* expression dropped about 75% in GC transfected with the siRNA targeting *Rgmb* when compared to a control siRNA (D, n = 6). Western blot with a phospho-Smad1/5/8 antibody (E, n = 4) and real-time PCR on *Id3* gene (G, n = 6) showed that the knowkdown of *Rgmb* does not affect AMH signalling pathway. Western blots were quantified and normalized from 4 experiments (F, n = 4). Data were analyzed using paired *t*-test. * *p*<0.05, ** *p*<0.01, *** *p*<0.001, a: *p* = 0.074. AMH response was not significantly different between control siRNA and *Rgmb* siRNA transfected GCs.

We then tested whether the BMP co-receptors RGMs could also be AMH co-receptors. We analysed the expression of *Rgma*, *Rgmb* and *Rgmc* in the ovary and primary GCs by RT-PCR (Fig. 6A) and q-PCR (Fig. 6B). *Rgma* and *Rgmc* expression was detected in total ovary but not in GCs (Fig. 6A and 6B). In GCs, we only detected *Rgmb* (Dragon) (Fig. 6A and 6B). In parallel, we confirmed the localization of RGMb in the ovary by immunohistochemistry where it is mainly present in GCs (Fig. 6C). To determine the potential role of RGMb in these cells, we transfected them with a siRNA directed against this co-receptor. We first checked whether this gene was down-regulated by q-PCR (Fig. 6D) and then analysed the sensitivity of these cells to AMH by Western blot using a phospho-Smad 1/5/8 antibody (Fig. 6E). siRNA transfection led to a 90% decrease of *Rgmb* mRNAs (Fig. 6D). The *Rgmb* knockdown GCs were as sensitive to AMH as control GCs (Fig. 6E and 6F). We then analysed the effect of AMH on *Id3* expression in GCs transfected with siRNA against *Rgmb* (Fig. 6G). AMH stimulation led to a 60% increased in *Id3* expression in GCs either transfected with control siRNA or not transfected. *Id3* also remained up-regulated by AMH at the same level in GCs transfected with siRNA against *Rgmb*. Altogether, these results showed that AMH can transduce its signal in the absence of RGMb indicating that this co-receptor was not essential for AMH signalling pathway in GCs.

### Smad1 and 5 are the main Smads used by AMH in GCs

**Figure 7:**
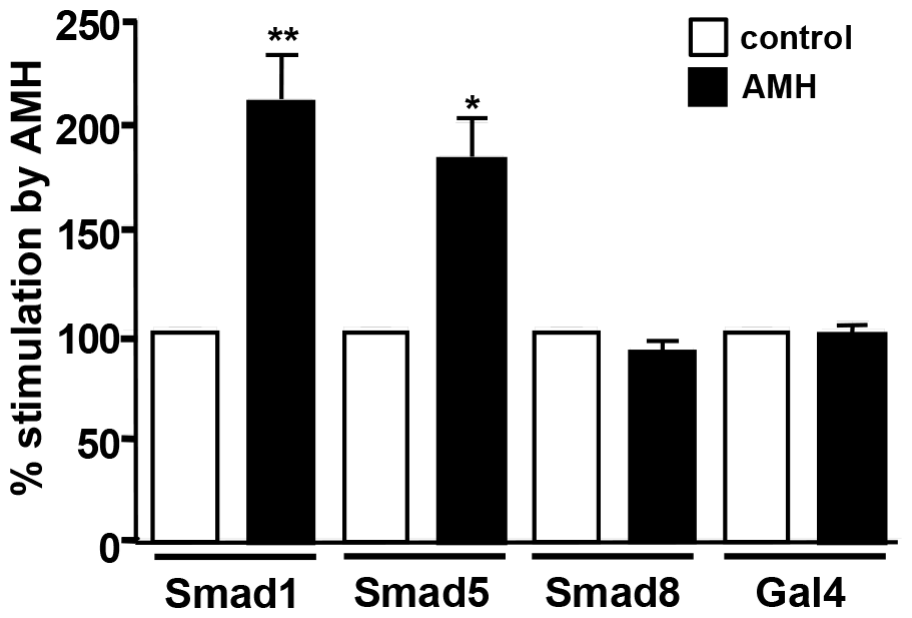
Smad1 and 5 are the main Smads activated by AMH in granulosa cells. GCs were co-transfected with a luciferase reporter construct (UAS-luc) and different expression constructs (Smad-Gal4). Four different expression constructs were transfected in combination with the reporter construct: Smad1-Gal4, Smad5-Gal4, Smad8-Gal4 and Gal4 as a control. Cells were stimulated (▪) or not (□) with 8 nM AMH. In the absence of AMH, the fusion protein Smad-Gal4 remained in the cytoplasm which is reflected by a basal expression level of luciferase. After 24h of treatment with AMH, the Smad-Gal4 protein was phosphorylated and could translocate into the nucleus and increase luciferase expression. Firefly luciferase activity measured in control medium was set to 100 arbitrary units. The results are expressed as a percentage of stimulation of Firefly luciferase activity measured in the presence of AMH (n = 3). Data were analyzed using paired *t*-test. ** *p*<0.01.

To investigate which Smad was important for AMH signalling in GCs, we first used siRNA for gene knockdown but we were not able to decrease Smad expression more than 50% (data not shown). We then used a reporter gene assay. 24 h after the preparation of GCs they were transfected with two plasmids: an expression plasmid which encodes a fusion protein (Smad1-Gal4-DBD/Smad5-Gal4-DBD or Smad8-Gal4-DBD) and a reporter plasmid which codes for a luciferase gene placed under the control of a promoter which contains UAS sequences (UAS-luc). These sequences are known to specifically bind Gal4 [36]. 24 h after transfection, the cells were treated during another 24 h with AMH (8 nM) and luciferase activity was measured (Fig. 7). As a control, we transfected Gal4-DBD with the reporter plasmid. As shown on Figure 7, AMH significantly increased Smad1 and Smad5 activity (110% and 80%, respectively) while it had no effect on Smad8. These results indicated that Smad1 and Smad5 were equally important for AMH signalling in GCs.

## Discussion

The aim of this study was to define the different actors of AMH signalling pathway including new target genes in immature GCs. We report that AMH up-regulates *Id3*, through BMPR-IA and that only Smad 1/5 are activated by AMH in these cells. We used in this study primary culture of mouse GCs and when available, we checked our results on GCs from conditional knockout mice for type I receptors.

GCs were prepared from 3 weeks old mouse immature ovaries. At this stage, ovaries are mainly composed of growing follicles expressing AMH and very few theca cells [22], [33]. As expected, immunocytochemistry showed that more than 90% of the cells express AMH, indicating that the culture predominantly contains GCs. Furthermore, these cultures expressed *Fshr* and *Inha* but not *Lhcgr*, a theca cell marker at this stage. The expression of *Fshr* is compatible with the presence of antral follicles at 3 weeks and with the fact that *Fshr* is detectable before the follicles become sensitive to FSH. These GCs were also properly transfected by siRNAs or plasmids allowing quantitative studies of the AMH signalling pathway. Finally, they expressed *Amhr2*, the AMH specific type II receptor, the three type I receptors and the R-Smads (Smad1 pathway) necessary to respond to AMH [37]. In keeping with this result, GCs in primary culture maintained their ability to respond to AMH by activating the Smad1 pathway.

We also addressed whether a new family of BMP co-receptors [18] the RGMs, RGMa, b (DRAGON) and c (Hemojuvelin), were expressed by GCs and could be AMH co-receptors. This hypothesis was based on the fact that BMPs and AMH share their signalling pathways, and that both RGMs and AMH are involved in anti-proliferative effects [38]–[43]. Indeed, *Rgmb* knockdown results in enhanced proliferation, adhesion, and migration in breast cancer cells [42] and in increased migration and invasion in PC-3 cells [43]. On the other hand, AMH has been described as a tumor suppressor gene in the mouse testis [41]. Moreover, *in vitro* studies have shown that AMH inhibits the growth of ovarian, endometrial and breast cancer cell lines [38]–[40]. We report that RGMb is the only RGM expressed in GCs but that the decrease of its expression using siRNA does not alter AMH responsiveness, indicating that RGMb is not essential for AMH signalling in GCs. Unfortunately, *Rgmb* knockout mice die within 2 to 3 weeks after birth suffering from immune and inflammatory disorders, precluding the study of the female reproductive tract [44].

We had previously shown in the adult mouse GC line AT29C-U493 that AMH activated the Smad1/5/8 signalling pathway [45]. Here we seek to identify which one of these Smad was preferentially phosphorylated and activated by AMH in GCs. We demonstrated, using reporter genes specific for each Smad, that only Smad1 and 5 are activated by AMH. Our results are in agreement with the phenotype of the transgenic models for Smad1/5/8. Indeed *Smad8* knockout mice are viable and fertile [46]–[48] and *Smad8* is the least expressed Smad in GCs indicating that this Smad is not important for ovarian physiology. In contrast, Smad1 and Smad5 function together in the ovary to suppress ovarian tumorigenesis [49]. Regarding the involvement of Smad proteins in AMH signalling, partial retention of Müllerian ducts is observed when *Smad5* is inactivated with or without another *Smad*, suggesting that *Smad5* is the most important Smad required for Müllerian ducts regression [16]. However, complete Müllerian duct retention in males occurs only when the three genes *Smad1*/*Smad5*/*Smad8* are conditionally inactivated [16]. Consistently, using siRNA against the different Smads, we were not able to decrease Smad expression more than 50% (data not shown).

We then studied which type I receptor was important in the AMH signalling pathway testing the ability of AMH to phosphorylate R-Smad 1/5/8 and to stimulate *Id3* expression in GCs either transfected with siRNA against *Acvr1*, *Bmpr1a* and *Bmpr1b*, or extracted from conditional KO mice for *Acvr1* or *Bmpr1a*. We did not consider *Acvrl1*, another type I receptor involved in R-Smad 1/5/8 pathway whose functional ligands are BMP9 and BMP10 [50]. Indeed, previous studies on AMH signalling in different cell types did not show the involvement of this receptor [13]–[15], [17], [51]–[53]. We showed that transfection of GCs with siRNA against *Bmpr1a*, prevents AMH to induce Smad1/5/8 phosphorylation and to regulate *Id3* expression. The use of corresponding conditional knockout mice, support these results. GCs isolated from *Bmpr1a* cKO mice ovaries do not transduce the AMH signal. These results indicate that BMPR-IA is the main AMH type I receptor in GCs. This is also the case for other AMH target cells. Indeed, BMPR-IA is necessary for AMH to mediate Müllerian duct regression since only *Bmpr1a* disruption induces Müllerian duct retention in mice [15]. Similarly, BMPR-IA is essential for AMH to activate Smad 1 in the Sertoli cell line SMAT-1 [17] and to induce Leydig cells differentiation in mice [53]. In keeping with a role of BMPR-IA in folliculogenesis, the majority of *Bmpr1a* cKO female mice are infertile due to a decrease of spontaneous ovulations and an inhibition of follicular development [24]. Concomitantly, 9 month-old *Bmpr1a* cKO mutant females exhibited increased follicular atresia [24]. Interestingly, the *Amhr2* KO mice also display a follicular atresia [54].

However, there is a redundancy among the type I receptors to transduce AMH effects. Indeed, Müllerian duct regression is blocked in about 50% of the conditional mutant males for *Bmpr1a* and occurs normally in 100% of the conditional mutant males for *Acvr1a*, but 100% of the males generated completely retained Müllerian duct derivatives only when both *Acvr1a* and *Bmpr1a* are conditionally inactivated, [16]. These findings indicate that BMPR-IA is the primary type I receptor required for Müllerian duct regression but that ActR-IA is capable of transducing the AMH signal in the absence of BMPR-IA [16]. Similarly, ActR-IA can compensate for the lack of BMPR-IA in SMAT-1 Sertoli cells [17]. Here we show that both GCs transfected with siRNA against *Acvr1* or isolated from *Acvr1* cKO mice ovaries are able to respond to AMH. Therefore, in contrast to Müllerian duct, Sertoli and Leydig cells, ActR-IA does not act as a secondary type I receptor for AMH in GCs. In addition, BMPR-IB does not have any compensatory effect in the absence of BMPR-IA. Interestingly, *Bmpr1b* KO mice are viable but females are infertile [55]. These females develop severe defects in cumulus expansion and insufficient uterine endometrial gland development. To complete our siRNA results, it would be interesting to study GCs isolated from *Bmpr1b* KO mice.

We needed some AMH target genes to validate the siRNA knockdown experiments. *Cyp19a1* and *Lhcgr* have been described as AMH target genes in a previous study [22]. However, we were unable to use them in the current study because their expression levels were too low in our GCs culture (data not shown), which is consistent with their stage of differentiation. Because AMH regulates genes encoding steroidogenic enzymes in Leydig and Sertoli cells namely *Hsd3b1* and *Cyp11a1* [20], [56], we have assayed the expression level of these genes after AMH exposure and could not detect any changes. Finally, since BMPs share some of their signalling pathway components with AMH, we tested some proven BMPs target genes. *Id* genes are BMP2 target genes in osteoblastic cells [57] and in the breast cancer cell line MCF-7 [58]. These proteins function as dominant negative basic helix loop helix (bHLH) transcription factors. They regulate many genes required for growth and differentiation, through the binding to E-box sequences located in the promoter of target genes [59]. *Id* genes are selectively up or down regulated depending on cell conditions [60]–[62]. In the ovine and hen GCs, the exposure to respectively BMP6 or BMP2 leads to an increase in all *Id* genes expression [60], [61]. Conversely, activin A has an inhibitory effect on *Id* gene expression [61]. Similarly, in porcine GCs expression levels of *Id2* and *Id3* are regulated by FSH and cumulus-oocyte-complex in an opposite way [62]. A recent study has demonstrated a functional relationship between the expression of all *Id* isoforms and the status of GC differentiation [25]. In our study, *Id3* gene expression is upregulated by AMH in immature GCs. Interestingly, *Lhcgr* expression is inversely correlated with *Id3* transcript level in hen undifferentiated GCs [25]. Because knockout studies have led to the conclusion that AMH plays an inhibitory role on follicle maturation [3], *Id3* might be one of the genes involved in this complex process.

In conclusion, using siRNA and transgenic mice for the different components of the AMH signalling pathway, we have shown that, like for the other AMH target cells, the most important type I receptor for AMH in GCs is BMPR-IA. Moreover, the main Smad proteins used by AMH in these cells are Smad 1 and Smad 5. Finally, we have identified a new AMH target gene in these cells, *Id3*, which could be involved in the effects of AMH on the differentiation of GCs and its other target cells.

## Supporting Information

Table S1 **Primers sequences used for mouse genotyping (Wt:wild-type allele, Mt:mutant allele).**
(DOC)

Table S2 **Primers sequences used for RT-PCR and real-time PCR.**
(DOC)
