# Supplementary material for: Anti-Müllerian Hormone Recruits BMPR-IA in Immature Granulosa Cells
Source: PLoS One. 2013 Nov 28;8(11):e81551. doi: 10.1371/journal.pone.0081551 (PMC3842941; doi:10.1371/journal.pone.0081551)
Supplement: Table S1 — Primers sequences used for mouse genotyping (Wt:wild-type allele, Mt:mutant allele). (DOC) [file pone.0081551.s001.doc]

| **Allele** | **Primers** | **Sequences (5'-3')** | **Size (bp)** | **Note** |
| --- | --- | --- | --- | --- |
| *Amhr2-Cre* | mAmhr2 E5-R | GAA-ACG-CAG-CTC-GGC-CAG-C | Mt :300 |  |
|  | PTMWbpA-F | CGC-ATT-GCT-TGA-GTA-GGT-GT |  |  |
| *Acvr1+/-* | mAlk2-F | ATG-CTA-GAC-CTG-GGC-AGC-CAT-A | Wt : 373  Mt : 337 |  |
|  | mAlk2-R | CAT-GCT-AGC-AGC-TCG-GAG-AAA-C |  |  |
|  | PGK | GAC-ACT-AGT-GAG-ACG-TGC-TAC-T |  |  |
| *Acvr1fx* | mAlk2fx-F | CCC-CCA-TTG-AAG-GTT-TAG-AGA-GAC | Wt : 250  Mt : 160+90 | *BglI* digest |
|  | mAlk2-R | CTA-AGA-GCC-ATG-ACA-GAG-GTT-G |  |  |
| *Bmpr1a+/-* | mAlk3fx3 | AGA-CTG-CCT-TGG-GAA-AAG-CGC | Mt : 190 |  |
|  | mAlk3fx5 | GGA-CTA-TGG-ACA-CAC-AAT-GGC |  |  |
| *Bmpr1afx* | mAlk3fx2 | GCA-GCT-GCT-GCT-GCA-GCC-TCC | Wt : 150  Mt : 230 |  |
|  | mAlk3fx4 | TGG-CTA-CAA-TTT-GTC-TCA-TGC |  |  |

**Table S1** Primers sequences used for mouse genotyping (Wt :wild-type allele, Mt :mutant allele)
