# Supplementary material for: Anti-Müllerian Hormone Recruits BMPR-IA in Immature Granulosa Cells
Source: PLoS One. 2013 Nov 28;8(11):e81551. doi: 10.1371/journal.pone.0081551 (PMC3842941; doi:10.1371/journal.pone.0081551)
Supplement: Table S2 — Primers sequences used for RT-PCR and real-time PCR. (DOC) [file pone.0081551.s002.doc]

| **Gene** | **Primers** | **Sequence (5'-3')** | **Size (bp)** | **Probe #** |
| --- | --- | --- | --- | --- |
| *Hprt* | Q-mHprt-474-s | TCA-ACG-GGG-GAC-ATA-AAA-GT | **91** | **22** |
|  | Q-mHprt-565-a | CCA-GTG-TCA-ATT-ATA-TCT-TCA-ACA-ATC |  |  |
| *Amhr2* | Q-mAmhr2-393-s | TGA-CTT-CTG-CAA-TGC-CAA-TTA | **129** | **16** |
|  | Q-mAmhr2-522-a | GAG-AAA-CAT-TCC-CAG-GAG |  |  |
| *Acvr1* | Q-mAlk2-978-s | AGG-GCT-CAT-CAC-CAC-CAA-T | **76** | **94** |
|  | Q-mAlk2-1053-a | GCC-ACT-TCC-TGA-TGT-ACA-CG |  |  |
| *Bmpr1a* | Q-mAlk3-875-s | CTC-ATT-TCC-ATG-GCT-GTC-TG | **95** | **78** |
|  | Q-mAlk3-969-a | CGA-CCC-CTG-CTT-GAG-ATA-CT |  |  |
| *Bmpr1b* | Q-mAlk6-837-s | GGA-GCA-GGA-CGA-GAC-ATA-CAT | **120** | **80** |
|  | Q-mAlk6-956-a | TGC-TTA-GCT-ATT-GTC-CTT-TGG-A |  |  |
| *Cyp11a1* | Q-mscc-1423-s | TGG-AGA-TGA-CCA-TCC-TCC-TTA | **72** | **67** |
|  | Q-mscc-1494-a | CCA-CAT-CAC-GGA-GAT-TTT-GA |  |  |
| *Fshr* | Q-mFSHR-398-s | ACC-CTG-AGG-CCT-TCC-AGA | **69** | **48** |
|  | Q-mFSHR-466-a | AGT-GTT-TAA-TGC-CTG-TGT-TGG-A |  |  |
| *Id3* | Q-mId3-724-s | GAG-GAG-CTT-TTG-CCA-CTG-AC | **128** | **19** |
|  | Q-mId3-851-a | GCT-CAT-CCA-TGC-CCT-CAG |  |  |
| *Inha* | Q-mINHA-405-s | GGA-AGA-TGT-CTC-CCA-GGC-TA | **61** | **33** |
|  | Q-mINHA-465-a | TGG-CTG-GTC-CTC-ACA-GGT |  |  |
| *Lhcgr* | mLHR-653-s | GAT-GCA-CAG-TGG-CAC-CTT-C | **127** | **31** |
|  | mLHR-779-a | GTA-GGA-TGA-CGT-GGC-GAT-G |  |  |
| *Rgma* | Q-mRGMa-s-UPL | CGC-CCT-ACA-CCT-AGT-CTT-CG | **99** | **19** |
|  | Q-mRGMa-a-UPL | GAT-TTT-GCA-GGG-GGA-GAT-G |  |  |
| *Rgmb* | Q-mRgmb-s | GAG-TTT-TGC-AAG-GCA-CTT-CG | **60** | **71** |
|  | Q-mRgmb-a | GCA-GGC-CTT-TGA-AGT-TCG |  |  |
| *Rgmc* | Q-mRGMc-s-UPL | GCT-TGA-CCT-CGG-GAA-ACA-T | **129** | **20** |
|  | Q-mRGMc-a-UPL | ACC-GGG-GAC-TAG-GGG-ACT |  |  |
| *Smad1* | Q-mSmad1-1370-s | AAC-ACC-AGG-CGA-CAT-ATT-GG | **76** | **91** |
|  | Q-mSmad1-1445-a | CAC-TGA-GGC-ATT-CCG-CAT-A |  |  |
| *Smad4* | Q-mSmad4-1858-s | CCG-TGG-GTG-GAA-TAG-CTC | **63** | **84** |
|  | Q-mSmad4-1920-a | GGT-CAT-CCA-CAC-CGA-TGC |  |  |
| *Smad5* | Q-mSmad5-1167-s | GCA-GTA-ACA-TGA-TTC-CTC-AGA-CC | **61** | **107** |
|  | Q-mSmad5-1227-a | GCG-ACA-GGC-TGA-ACA-TCT-C |  |  |
| *Smad8* | Q-mSmad8-389-s | ACC-ATT-ACC-GCA-GAG-TGG-AG | **73** | **16** |
|  | Q-mSmad8-461-a | TGA-GGG-TTG-TAC-TCG-CTG-TG |  |  |
| *Star* | Q-mStar-312-s | TTG-GGC-ATA-CTC-AAC-AAC-CA | **65** | **11** |
|  | Q-mStar-376-a | ACT-TCG-TCC-CCG-TTC-TCC |  |  |

**Table S2** Primers sequences used for real-time PCR
